# Nicotine supplementation enhances simulated game performance of archery athletes

**DOI:** 10.1186/s12970-021-00413-9

**Published:** 2021-02-18

**Authors:** Bao-Lien Hung, Li-Jung Chen, Yi-Ying Chen, Jhih-Bang Ou, Shih-Hua Fang

**Affiliations:** 1 Department of Sports Medicine, China Medical University, Taichung, 40402 Taiwan; 2 Department of Exercise Health Science, National Taiwan University of Sport, Taichung, 40404 Taiwan; 3 Institute of Athletics, National Taiwan University of Sport, No. 16, Section 1, Shuang-Shih Road, Taichung, 40404 Taiwan

**Keywords:** Nicotine, Cognitive abilities, Archery performance

## Abstract

**Background:**

Nicotine is beneficial to mood, arousal and cognition in humans. Due to the importance of cognitive functioning for archery athletes, we investigated the effects of nicotine supplementation on the cognitive abilities, heart rate variability (HRV), and sport performance of professional archers.

**Methods:**

Eleven college archers were recruited and given 2 mg of nicotine supplementation (NIC group) and placebo (PLA group) in a crossover design.

**Results:**

The results showed that at 30 min after the intake of nicotine gum, the “correct rejection” time in the NIC group was significantly lower than that of the PLA group (7.29 ± 0.87 vs. 8.23 ± 0.98 msec, *p* < 0.05). In addition, the NIC group completed the grooved pegboard test in a shorter time than the PLA group (48.76 ± 3.18 vs. 53.41 ± 4.05 s, *p* < 0.05), whereas motor reaction times were not different between the two groups. Saliva α-amylase activity was significantly lower after nicotine supplementation (*p* < 0.01) but increased immediately after the archery test in the NIC group (*p* < 0.05). In addition, nicotine supplementation significantly decreased HRV and increased the archery score (290.58 ± 10.09 vs. 298.05 ± 8.56, *p* < 0.01).

**Conclusions:**

Nicotine enhances the performance of archery athletes by increasing cognitive function and stimulating the sympathetic adrenergic system.

## Introduction

Athletes, especially in professional team/strength sports (e.g., baseball, ice hockey, wrestling, gymnastics), have different motivations for tobacco consumption, such as enhancing concentration, helping relaxation, allaying fatigue or improving performance [1–4]. However, an association between long-term cigarette smoking and reduced physical performance of athletes has been reported [5]. There is a strong body of evidence related to the harms of tobacco use to lung health from direct and passive exposure to tobacco smoke [6]. Pulmonary function was reduced in active smokers in comparison with nonsmokers, especially in skill and power sports [7]. Nicotine, the most abundant alkaloid constituent in tobacco [8], is currently not on the World Anti-Doping Agency (WADA) prohibited list. The use of smokeless tobacco has thus been recommended to obtain the beneficial effects of nicotine and avoid the negative effect of tobacco smoke on the respiratory tract [9].

Nicotine, one of the most widely consumed psychostimulants [10], activates the sympathetic nervous system (SNS) and can increase blood pressure and catecholamine concentrations [11]. Nicotine diffuses readily into the brain and binds to nicotinic cholinergic receptors (nAChRs), which are ligand-gated ion channels [12]. A meta-analysis study reported significant positive effects of nicotine in six domains of cognitive function, including fine motor abilities, alerting attention-accuracy and response time (RT), orienting attention-RT, short-term episodic memory-accuracy and working memory-RT [13]. In addition, nicotine enhanced cognitive performance in both smokers and nonsmokers [14]. Moreover, nicotine exhibited promising effects in improving the cognitive functions of patients with pathological diseases such as Parkinson’s disease, Alzheimer’s disease, schizophrenia and attention-deficit/hyperactivity disorder [15].

Additionally, a systemic review [16] reported that nicotine increased heart rate (HR) in most studies, reflecting an upregulation in sympathetic activity [17], but decreased heart rate variability (HRV), which is an index of autonomic function [18] and is often used as a noninvasive means to assess cardiac autonomic activity in athletic populations [19, 20]. The low frequency (LF) and high frequency (HF) components of HRV are considered markers of sympathetic and parasympathetic nerve activities [21].

Regarding autonomic regulation, simultaneous and opposite changes in sympathetic and parasympathetic activity of athletes are dependent on several individual and environmental factors, such as training intensity, exercise types and mental stress [22–24]. The regulation of sympathetic and parasympathetic activities is very important for recovery and energy homeostasis during and after exercise in athletes [25]. As another marker of SNS activity, salivary α-amylase (sAA) was elevated under physical (e.g., exercise) [26] and psychological (e.g., examination) stress and reflected blood catecholamine levels [27]. Furthermore, a positive correlation between sAA and the LF/HF ratio was reported as a surrogate for sympathetic tone [28, 29]. Correlations of sAA with anxiety and game records in archery competition have been reported [30]. To supplement HRV data with a neuroendocrinological approach, sAA has attracted much attention as a biomarker of activation of the autonomic nervous system [27].

Based on recent reviews, the effects of nicotine or smoking on physical performance in different types of exercise have not been consistent. Some studies reported ergogenic effects, whereas other studies found unaltered or ergolytic effects [16, 31]. Recurve archery is an Olympic sport that requires attention, fine movement control, upper body strength and endurance [32, 33]. Nicotine was shown to have a positive effect on attention and facilitate the focus of cognitive responses on a specific task [34, 35]. In addition, nicotine increased skeletal muscle contraction force and delayed fatigue via activation of central cholinergic receptors and peripheral sympathoadrenal effects as an enhancer of power-based sporting performance [31, 36]. Therefore, we hypothesized that nicotine supplementation might be helpful for archery performance.

Several studies have investigated the effects of various dosing forms of nicotine, including transdermal patches, oral smokeless tobacco (snus), oral nicotine gum and inhalers [31]. According to the administration instructions for nicotine gum, one tablet (containing 2 mg of nicotine) is recommended for the first time. Because all the participants recruited in this study were nonsmokers, the reported lowest effective dose of nicotine (i.e., 2 mg) [31, 37, 38] was administered. Therefore, the aim of this study was to investigate the effects of 2-mg nicotine gum on cognition, neuroendocrinological responses (HRV and sAA) and archery performance in professional athletes.

## Methods

### Participants

Eleven healthy, male nonsmokers were recruited in 2018 through printed advertisements and by word of mouth from archery sport teams of both the National Taiwan University of Sport and the National Chung Cheng University in Taiwan. The participants were required to meet the following criteria: (1) they were archers who used a recurve bow; (2) their archery performances were at the national level; and (3) they had been continually training for a minimum of 2 h ≥ three times per week for at least 3 years. Participants were excluded if they had cardiac disease histories, were injured or were unable to participate in normal training (*n* = 0). Therefore, the recruited archers were all qualified and included in this study (*n* = 11). The sample size was calculated by G*power 3.1.9.4 (available at http://www.gpower.hhu.de). One of our aims was to evaluate the effects of nicotine use on HRV using two-way repeated measures analysis of variance (ANOVA) (within factors). Type 1 error (alpha) was set at the level of 5% (*p* = 0.05), and the power was set at 80%. The effect size f was determined by partial *eta squared* (*η*_*p*_^*2*^) based on previous research (*η*_*p*_^*2*^ = 0.34) [31]. The results showed that at least 6 participants were required; thus, the sample size in this study was sufficient. The mean age of the participants was 20.3 ± 0.3 years, the mean body weight was 71.1 ± 9.2 kg, and the mean body height was 174.4 ± 4.5 cm. All participants were advised to abstain from stimulants such as coffee or energy drinks for 12 h. Each participant was fully informed of all potential risks and experimental procedures, after which informed written consent was obtained. All experimental procedures and protocols were approved by the Institutional Human Ethics Committee of Jen-Ai Hospital, Taichung, Taiwan.

### Experimental protocol and measures

**Fig. 1:**
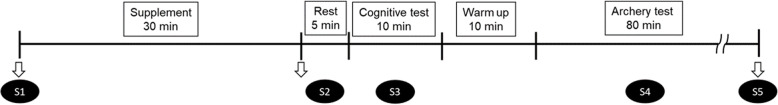
Graphical representation of the experimental protocol. Stage 1 (S1): before supplementation; S2: after supplementation and before cognitive testing; S3: during cognitive testing; S4: during the archery test; S5: immediately after the archery test. The arrow signs represent the time points when saliva was collected

The experimental protocol (schematized in Fig. 1) was a randomized, placebo-controlled crossover design, which was conducted across a period of 1 week. On arrival at the laboratory, the participants were fitted with a heartrate strap and monitor (Polar V800, Polar Electro Oy, Kempele, Finland) and seated for 5 min, after which baseline HR measurements were individually taken in stages 1–5. First, they were asked to remain in the seated position, and saliva samples were collected (stage 1, S1). For experimental trials, the participants chewed 2-mg nicotine gum (NIC group) or flavor-matched placebo gum (PLA group) for 30 min while seated, followed by the participants’ saliva being collected again (stage 2, S2). Then, they completed cognitive tests (Vienna Test System (VTS)–Cognitrone test (COG) and the grooved pegboard test (GPT)) (stage 3, S3) [39]. Furthermore, their archery performance was measured in a simulated game (stage 4, S4). Then, saliva samples were collected (stage 5, S5). Experimental trials were conducted at the same time of day, and the day of and prior to any experimental trial was marked by abstinence from alcohol, any exercise and only habitual caffeine use (as abstinence would in itself have confounding withdrawal-related effects). Additionally, the participants were asked to replicate their diet during the first experimental visit for subsequent trials to ensure a similar metabolic state.

### Nicotine intervention

The participants were instructed to consume the gum according to the manufacturer’s recommendations: one piece of 2-mg mint-flavored nicotine gum or placebo gum (Nicorette Icy Mint, Johnson & Johnson Pacific, Auckland, New Zealand) was introduced into the mouth followed by the instructions “chew until there is a strong taste, then place between your cheek and gums, and chew again when the taste has faded”; this stage lasted for 30 min.

### Cognitive function assessments

All participants completed the cognitive outcome measures including the VTS–Cognitrone test (COG) and grooved pegboard test (GPT). The COG test measures attention and concentration through the comparison of figures regarding their congruence on a computer screen. The participants were presented with an abstract figure, and they were asked to match the figure to a model. The mean time for “correct rejections” made within the total working time of 7 min was recorded [39]. The GPT is a test of manual dexterity, speed of processing, and hand-eye coordination, which has been used in previous studies [39, 40]. The GPT includes 25 holes with randomly positioned slots. Participants were asked to put the pegs in the board in a fixed order and in the correct direction with only one hand being used. They were encouraged to perform the task as quickly as possible. The total time to completion was recorded in seconds. In this study, each participant was tested twice with his dominant and nondominant hands. Then, the average time was calculated to give an overall score.

### Archery test

The performance for the archery assessment was assessed according to the rules of national archery competition in outdoor areas. The participants used their own bow and arrow equipment. Before the experiment, they all had adequate warm-up and corrected the sighting position. Briefly, each player fired 72 arrows, 36 arrows for a game. In each single day, they had 2 games. Every participant fired six arrows for a round, and each round time limit was 4 min. Each arrow was worth up to 10 points; therefore, a single game overall had a possible score of 360 points. They rested for 15 min after the end of the first game. After the end of each round, the arrows were pulled, and the same person recorded the scores to verify the accuracy. Any guidance was excluded during the exercise. The same archery test was performed on three different days in a week to obtain the average archery assessment in each group.

### Heart rate variability measurements

Beat-to-beat heart rate was recorded with 1-ms resolution using portable HR monitors. The HR, percentage of differences between adjacent normal RR intervals of more than 50 ms (pNN50), low-frequency normalized units (LFnu) and high-frequency normalized units (HFnu) were calculated from the 5-min recordings. LF/HF was calculated from the ratio of LFnu over HFnu. Spectral analysis was performed by the maximum entropy method, and autoregressive coefficients were estimated using the Burg algorithm. The power spectrum was calculated from 0.01 to 0.40 Hz with 0.01-Hz frequency resolution. LF and HF components were calculated through integration of the power spectra of 0.04 to 0.15 Hz and 0.15 to 0.40 Hz, respectively.

### Saliva collection and assay

Unstimulated whole saliva was collected as previously described [41]. Briefly, all participants were seated and asked to thoroughly rinse their mouth with 30 ml of sterile distilled water before sample collection. The participants remained seated for 10 min until all saliva samples were collected into sterile plastic containers. Two ml saliva samples were collected at three time points: before gum administration (S1), after gum administration (S2), and after the archery test (S5). The saliva samples were immediately stored at − 80 °C until assay. Since the half-life of nicotine is short, cotinine, the major metabolite, was used as a reliable marker based on its longer half-life. The salivary cotinine level was determined using an enzyme-linked immunosorbent assay (ELISA) kit (Cozart Bioscience Ltd., Oxfordshire, UK). α-Amylase activity was determined using a kinetic reaction assay kit (Salimetrics LLC, State College, PA, USA) according to the manufacturer’s instructions. All samples were measured in triplicate. The intra-assay coefficients of variation (CVs) for the measurements of cotinine and α-amylase activity were 5 and 4%, respectively.

### Statistical analyses

All data are expressed as the mean ± standard deviation (SD). Two-way (condition x time) repeated measures ANOVA with a Tukey post hoc test was used to determine differences between the conditions and time points. Statistical comparisons between the NIC and PLA groups were analyzed using paired t-tests. *Partial eta squared* (*η*_*p*_^*2*^) values are reported as a measure of effect sizes, with demarcations of small (< 0.09), medium (> 0.09 and < 0.25) and large (> 0.25) effects [31]. Significant differences were set at *p* < 0.05.

## Results

All participants completed the study without reporting negative side effects. The mean salivary cotinine level at 30 min after nicotine supplementation was 10.3 ± 1.8 ng/ml and undetectable at S1 and S5.

### Effect of nicotine on cognitive and archery performance

Thirty minutes after chewing the nicotine gum, the participants performed the cognitive function tests. The mean “correct rejection” time in the NIC group was significantly shorter than that in the PLA group (7.29 ± 0.87 vs. 8.23 ± 0.98 msec, respectively, *p* = 0.038; Table 1), indicating that the NIC group had better concentration than the PLA group. In addition, the NIC group completed the GPT in a significantly shorter time than the PLA group (48.76 ± 3.18 vs. 53.41 ± 4.05 s, respectively, *p* = 0.003). These findings indicated that the NIC group had better cognitive performance than the PLA group. The participants’ archery performance was measured by calculating the mean score of 36 arrows for each game from three independent days. Each arrow was worth up to 10 points. The archery score in the NIC group was significantly higher than that of the PLA group (298.05 ± 8.56 vs. 290.58 ± 10.09, respectively, *p* = 0.009; Table 1).

**Table 1:** Cognitive function and archery performance of each group

Parameter	PLA group			NIC group			*P* value
Concentration							
Mean time “Correct rejection” (msec)	8.23	±	0.98	7.29	±	0.87^#^	0.038
Speed of processing (sec)	53.41	±	4.05	48.76	±	3.18^##^	0.003
Archery score	290.58	±	10.09	298.05	±	8.56^##^	0.009

Values are presented as the mean ± SD

*PLA group* placebo group, *NIC group* nicotine group

^#^*p* < 0.05, ^##^*p* < 0.01 compared to the PLA group

### Effect of nicotine on heart rate variability

After supplementation with nicotine (S2), the HR, LF and LF/HF ratio were significantly increased, whereas the pNN50 and HF significantly decreased and were lower than baseline (S1) (Table 2). When the participants were taking the cognitive function test (S3), all the HRV parameters were similar to those at baseline in both groups. During archery performance (S4), the HR, LF and LF/HF ratio were significantly higher, whereas the pNN50 and HF were significantly lower than their own baseline in both groups. In particular, HR in the NIC group was significantly higher than that in the PLA group during the archery test. All HRV parameters in both groups, with the exception of HR in the NIC group returned to baseline after the archery test (S5).

**Table 2:** The heart rate variability in both groups at different stages

Outcomes	Condition	Time					Condition effect		Time effect		Condition x Time	
		S1	S2	S3	S4	S5	*p*	η_p_^2^	*p*	η_p_^2^	*p*	η_p_^2^
HR (bpm)							.028	.474	<.001	.815	.039	.319
	PLA	82.23 ± 6.50	82.50 ± 6.83	79.75 ± 5.96	95.41 ± 5.33^aaa^	81.72 ± 6.85						
	NIC	79.51 ± 5.15	84.49 ± 6.85^a^	84.24 ± 8.14	101.70 ± 8.04^aaa,##^	87.85 ± 9.53^a^						
pNN50 (%)							.229	.175	<.001	.562	.023	.290
	PLA	14.16 ± 7.83	13.26 ± 9.94	18.37 ± 10.05	5.56 ± 3.63^aa^	17.03 ± 9.56						
	NIC	18.86 ± 9.94	8.77 ± 5.16^aa^	13.08 ± 6.40	5.25 ± 2.82 ^aa^	12.21 ± 8.21						
LF(n.u.)							.371	.081	.008	.396	.588	.067
	PLA	68.30 ± 7.22	69.41 ± 7.55	64.62 ± 8.00	77.39 ± 12.74^a^	61.63 ± 11.14^a^						
	NIC	65.56 ± 12.90	72.01 ± 8.11^a^	68.33 ± 9.10	77.52 ± 10.05 ^aa^	65.35 ± 10.56						
HF(n.u.)							.371	.081	.008	.396	.588	.067
	PLA	31.70 ± 7.22	30.59 ± 7.55	35.38 ± 8.00	22.61 ± 12.74^a^	38.37 ± 11.14^a^						
	NIC	34.44 ± 12.90	27.99 ± 8.11^a^	31.67 ± 9.10	22.48 ± 10.05^aa^	34.65 ± 10.56						
LF/HF							.655	.021	.002	.565	.516	.065
	PLA	2.44 ± 0.75	2.61 ± 0.81	2.14 ± 0.91	5.28 ± 3.31^a^	2.02 ± 1.00						
	NIC	2.26 ± 0.98	3.15 ± 1.17^a^	2.70 ± 1.58	4.73 ± 2.04^aa^	2.33 ± 0.83						

Values are presented as the mean ± SD

*η*_*p*_^*2*^ Partial eta squared

^a^ *p* < 0.05, ^aa^ *p* < 0.01, ^aaa^ *p* < 0.001 compared to S1 in each group

^##^*p* < 0.01 compared to the PLA group

### Effect of nicotine on saliva α-amylase activity

**Fig. 2:**
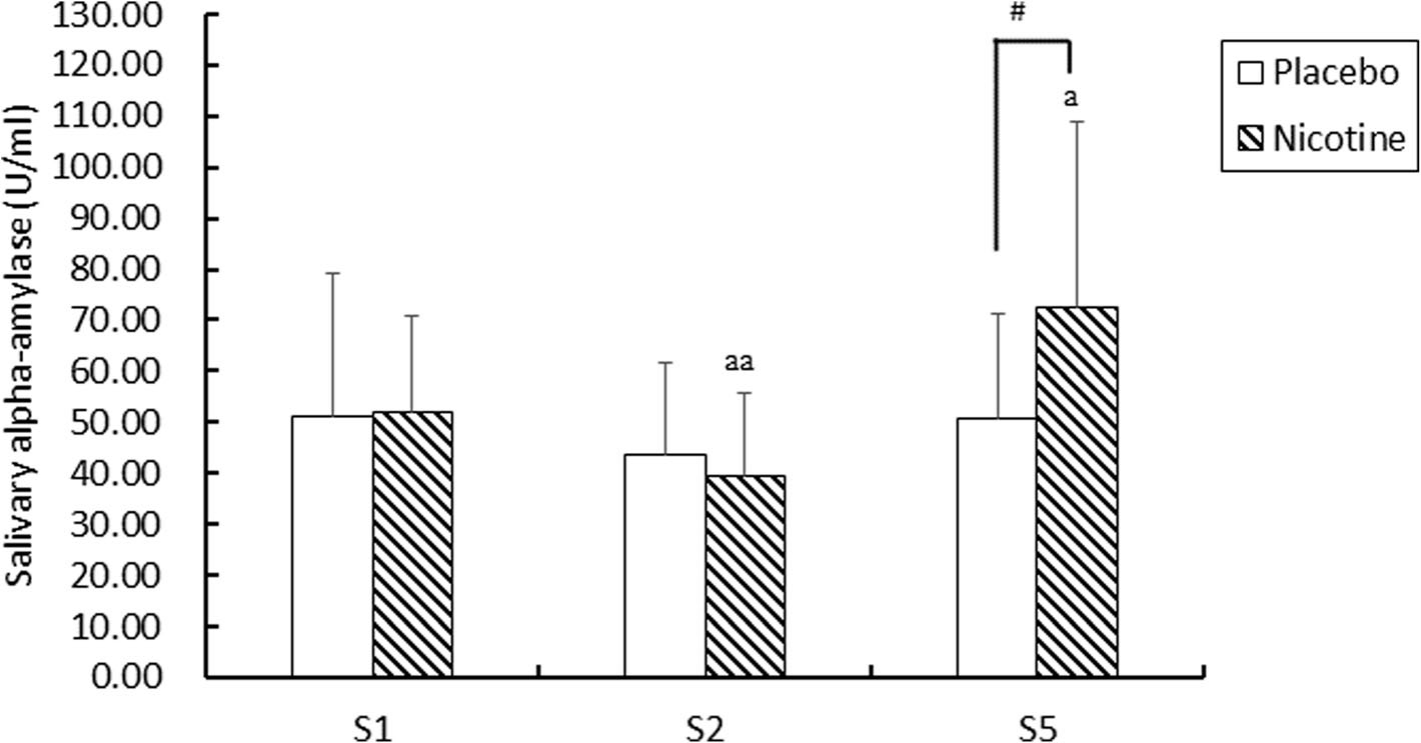
Salivary α-amylase activity in Stage 1 (S1), S2 and S5. Values are presented as the mean ± SD. Stage 1 (S1): before supplementation; S2: after supplementation and before cognitive testing; S5: immediately after the archery test. a: *p* < 0.05; aa: *p* < 0.01 compared to S1 in each group, #: *p* < 0.01, compared to the placebo group

Saliva α-amylase activity was significantly decreased (39.64 ± 15.97 vs. 51.82 ± 19.09 U/ml, *p* < 0.01) after nicotine supplementation (S2) but increased immediately after the archery test (S5) and was higher than the basal level (S1) (72.36 ± 36.66 vs. 51.82 ± 19.09 U/ml, respectively, *p* < 0.05; Fig. 2). At S5, the NIC group showed significantly higher α-amylase activity than the PLA group (72.36 ± 36.66 vs. 50.74 ± 20.44 U/ml, respectively, *p* < 0.01). There was no difference at the three time points in the PLA group.

## Discussion

To our knowledge, our study was the first to investigate the acute effects of 2-mg nicotine gum supplementation on cognition, HRV, sAA and exercise performance in archery athletes. We found that nicotine supplementation enhanced cognitive function by shortening the time for correct rejection and time on the grooved pegboard test. Second, the HR, LF and LF/HF ratio were increased, whereas the pNN50 and HF were decreased. Third, sAA was increased during the exercise stage. Last, the archery score was significantly increased. The results suggested that there was a positive relationship between nicotine supplementation and archery performance.

In this study, the results showing that nicotine shortened the time for correct rejection and time on the grooved pegboard test were in good agreement with a previous study reporting that the performance of baseball players on math and Stroop tests was better in smokeless tobacco users than nonusers, but smokeless tobacco did not influence psychomotor tasks (choice reaction time and anticipation time) [41]. Consistently, an acute effect of 4-mg nicotine administration (independent of smoking history) was reportedly related to the reaction time on the two-letter search task [42].

Our results demonstrating an increase in the HR, LF and LF/HF ratio were consistent with previous studies reporting that nicotine decreased HRV by stimulating the sympathetic adrenergic system [43, 44] and increased HR during submaximal exercise [45], as well as increased LF/HF ratio and HR during training in athletes [46]. sAA was increased immediately after the archery test, which was in good agreement with previous studies reporting that sAA was increased 3 min before and 10 min after archery competition [30] and when sympathetic nerves were activated [47].

Some athletes feel that smokeless tobacco could enhance their performance [13, 41, 48], but others do not believe so [49, 50]. That the response to nicotine was significantly heterogeneous among individuals might be due to multiple reasons, including genetic factors, receptor availability, gender, absorption, and performance as well as neural activations at baseline or under placebo [51]. One review [16] identified only two of six studies reporting significant improvements in exercise performance. One study reported that subjects with a 7-mg transdermal nicotine patch given 12 h prior to a 75% VO_2_max cycling exercise resulted in an increased exercise time by 17 ± 7% [52] but no effect on the rate of perceived exertion. Another study reported in healthy team sports members who had never smoked that chewing 2-mg nicotine gum 20 min prior to exercise caused higher HR and increased leg extensor force but did not affect countermovement jump height or Wingate anaerobic capacity [31]. In addition, a recent study demonstrated that 5-mg nicotine strip administration increased repeated anaerobic performance in peak power and average power in nicotine-naïve athletes [53]. This result indicated that the probable mechanism of nicotine might be through activation of the SNS and increasing heart rate as well as blood pressure [53]. After 12 h of overnight nicotine abstinence, snus (approximately 8 mg nicotine) induced an increase in time to exhaustion in a 80% VO_2_max exercise test [54]. In addition, the muscular and cerebral oxygenation in nontobacco users increased with snus (8 mg nicotine) administration in a 65% of the maximal aerobic power output exercise until exhaustion test, but fatigue perception and time to exhaustion were not affected [55].

In this study, archery scores were significantly increased by nicotine supplementation. Archery demands very specific muscle strength, endurance fitness, and hand-eye coordination for successful intermittent repetitive shooting performance [56, 57]. All these characteristics of archers are modulated by the autonomic nervous system [58]. A previous study demonstrated that 6-mg nicotine gum enhanced visuospatial selective attention with regard to early visual encoding and analysis in nonsmokers [59]. In addition, 2-mg nicotine gum reduced movement times, increased velocities and resulted in more fluent handwriting movements in nondeprived smokers and smokers [38], and increased cutaneous blood flow and skin temperature [37]. Furthermore, a study demonstrated that intravenous injections of 0.4 to 1 mg of nicotine to nonsmokers caused an increase in blood flow in muscle [60]. Taken together, increased blood flow to supply adequate oxygen to active skeletal muscle might be one of the reasons explaining the enhanced archery performance.

The athletes believed that consumption of nicotine/nicotine-containing substances proved ergogenic through augmenting saliva secretion, stimulating satiety, improving reaction time and concentration, helping relaxation and producing desirable arousal and attention [49, 61]. In relation to the archery performance test, we monitored autonomic assessment for the spectral analysis of HRV and sAA activity. Based on our results, we suggest that nicotine might help athletes maintain concentration and sympathetic activation, thereby improving sport performance. However, our study has several limitations. First, because of the limited number of male national-level archery athletes using the recurve bow, the sample size of this study was small. Whether similar effects would occur with other types of exercise needs more investigation. Second, we did not monitor female archers or archers who were smokers. It is unclear whether our findings regarding the acute effects of nicotine can be applied to other populations. Additionally, both the pharmacokinetics and pharmacodynamics of nicotine can differ between smokers and nonsmokers. Third, we used a single, low dose of nicotine in this study. Therefore, the dose-dependent effect of nicotine on exercise performance needs further study. Moreover, if athletes use nicotine too often, it is necessary to examine whether there is a decrease in the ergogenic effect as has been seen with caffeine use.

Tobacco use is highly addictive and a major risk factor for cardiovascular and respiratory diseases, over 20 different types or subtypes of cancer, and many other debilitating health conditions. More than 8 million people die from tobacco use each year [62]. Previous studies have indicated that nicotine exhibits dose-dependent bidirectional regulation of mouse [63] and rat [64] stem cell proliferation. Moreover, long-term and/or continuous nicotine administration has been reported to have a deleterious effect on ischemic brain injury [65]. Since nicotine has the potential to be additive, there is a possibility that athletes who are nonusers of tobacco may become addicted. Previous studies using questionnaires showed that nicotine gum is less addictive than tobacco cigarettes [66], and addiction to nicotine gum in never-smokers is probably quite rare [67]. In addition, the use of 2-mg nicotine gum as nicotine replacement therapy for 5 years appeared to be safe and to not cause cancer [68, 69]. However, the usage of nicotine still needs to be carefully considered because it is a psychoactive and addictive substance with effects in the brain. In the future, there is a need for more research examining the effects of different dosages of nicotine on cognitive functions, HR, LF and LF/HF ratios between smokers and nonsmokers to evaluate nicotine as a potential ergogenic aid for different sports.

## Conclusions

In summary, these results indicated that 2-mg nicotine gum supplementation enhanced cognitive function, decreased saliva α-amylase activity and HRV through stimulating the sympathetic adrenergic system. More importantly, the archery scores were significantly increased after nicotine supplementation.

## Data Availability

The datasets used and/or analyzed during the current study are available from the corresponding author on reasonable request.

## Abbreviations

HRV: Heart rate variability
NIC: Nicotine
PLA: Placebo
WADA: World Anti-Doping Agency
SNS: Sympathetic nervous system
nAChRs: nicotinic cholinergic receptors
RT: Response time
HR: Heart rate
LF: Low frequency
HF: High frequency
sAA: salivary α-amylase
ANOVA: Analysis of variance
VTS: Vienna Test System
COG: Cognitrone test
GPT: Grooved pegboard test
pNN50: percentage of differences between adjacent normal heart rate intervals of more than 50 ms
LFnu: Low-frequency normalized units
HFnu: High-frequency normalized units
CV: Coefficient of variation
SD: Standard deviation
